# Niacin Improves Intestinal Health through Up-Regulation of AQPs Expression Induced by GPR109A

**DOI:** 10.3390/ijms23158332

**Published:** 2022-07-28

**Authors:** Shilong Liu, Yueqin Qiu, Fang Gu, Xiaoming Xu, Shansen Wu, Zhenhao Jin, Li Wang, Kaiguo Gao, Cui Zhu, Xuefen Yang, Zongyong Jiang

**Affiliations:** State Key Laboratory of Livestock and Poultry Breeding, Key Laboratory of Animal Nutrition and Feed Science in South China, Ministry of Agriculture and Rural Affairs, Guangdong Provincial Key Laboratory of Animal Breeding and Nutrition, Maoming Branch, Guangdong Laboratory for Lingnan Modern Agriculture, Institute of Animal Science, Guangdong Academy of Agricultural Sciences, Guangzhou 510640, China; liushilong94@126.com (S.L.); qiuyueqin87@126.com (Y.Q.); gf13633778817@163.com (F.G.); xm20130613@163.com (X.X.); wushansen@stu.scau.edu.cn (S.W.); zhenhaojin1@163.com (Z.J.); wangli1@gdaas.cn (L.W.); gaokaiguo@gdaas.cn (K.G.); juncy2010@gmail.com (C.Z.); jiangzy@gdaas.cn (Z.J.)

**Keywords:** piglets, IPEC-J2 cell, niacin, aquaporins, diarrhea, GPR109A

## Abstract

(1) Background: Changes in the expression of aquaporins (AQPs) in the intestine are proved to be associated with the attenuation of diarrhea. Diarrhea is a severe problem for postweaning piglets. Therefore, this study aimed to investigate whether niacin could alleviate diarrhea in weaned piglets by regulating AQPs expression and the underlying mechanisms; (2) Methods: 72 weaned piglets (Duroc × (Landrace × Yorkshire), 21 d old, 6.60 ± 0.05 kg) were randomly allotted into 3 groups for a 14-day feeding trial. Each treatment group included 6 replicate pens and each pen included 4 barrows (*n* = 24/treatment). Piglets were fed a basal diet (CON), a basal diet supplemented with 20.4 mg niacin/kg diet (NA) or the basal diet administered an antagonist for the GPR109A receptor (MPN). Additionally, an established porcine intestinal epithelial cell line (IPEC-J2) was used to investigate the protective effects and underlying mechanism of niacin on AQPs expression after Escherichia coli K88 (ETEC K88) treatment; (3) Results: Piglets fed niacin-supplemented diet had significantly decreased diarrhea rate, and increased mRNA and protein level of ZO-1, AQP 1 and AQP 3 in the colon compared with those administered a fed diet supplemented with an antagonist (*p* < 0.05). In addition, ETEC K88 treatment significantly reduced the cell viability, cell migration, and mRNA and protein expression of AQP1, AQP3, AQP7, AQP9, AQP11, and GPR109A in IPEC-J2 cells (*p* < 0.05). However, supplementation with niacin significantly prevented the ETEC K88-induced decline in the cell viability, cell migration, and the expression level of *AQPs* mRNA and protein in IPEC-J2 cells (*p* < 0.05). Furthermore, siRNA GPR109A knockdown significantly abrogated the protective effect of niacin on ETEC K88-induced cell damage (*p* < 0.05); (4) Conclusions: Niacin supplementation increased AQPs and ZO-1 expression to reduce diarrhea and intestinal damage through GPR109A pathway in weaned piglets.

## 1. Introduction

Originally, the term Aquaporins (AQPs) referred to a group of integral membrane proteins that have a higher coefficient of water [1,2]. Until now, 13 isoforms of AQPs have been identified in mammals. And 11 isoforms of AQPs (AQP1–11) have been found to be present in the gastrointestinal tract. Depending on a special structure named the aromatic/arginine selectivity filter (ar/R SF), the AQP1, 2, 4, 5, 6, and 8 were shown to be selectively permeable to water, AQP3, 7, 9, and 10 are permeable to glycerol, urea, and other small solutes as well as water, and AQPs11 and 12 are known as super-aquaporins, and their functions are still being explored [3,4,5,6]. AQPs also maintain the balance of intestinal fluid movement and the homeostasis of the internal and external environment in the mammalian intestine [7,8]. Recently, increasing evidence has shown that AQPs regulate cell proliferation and migration and are involved in intestinal inflammation [9,10,11]. Apart from that, based on the important role played by AQP in intestinal function and/or fluid homeostasis, it provides a direction for future research on diseases involving disruption of intestinal homeostasis (e.g., inflammatory bowel disease and irritable bowel syndrome) [8,12,13]. Hence, altered changes in localization and expression of AQPs may have an effect on diarrhea. In the ETEC K88 and bile acid diarrhea model, the expression of AQP3, AQP7, AQP8, AQP9, and AQP11 were significantly decreased in the gut [14,15]. However, it is not known whether niacin plays a role in regulating AQP expression.

Diarrhea is a major challenge for post-weaning piglets, and ETEC is the main factor causing post-weaning diarrhea in piglets, which could induce fluid losses, and impair the integrity of the intestinal epithelial mucosa and intestinal barrier function [16,17]. Weaning stress induced by changes in diet, environment, and an incompletely developed and functioning intestine, normally caused serious diarrhea and death of piglets, resulting in a great economic loss to swine production. A direct manifestation of diarrhea is increased water content in the feces, which in turn indicated an impaired intestinal function of water absorption [18]. AQPs located in the intestinal epithelium has been shown to play a critical role in modulating intestinal absorption of water through the transport of ions and water molecules [19]. Additionally, previous studies have demonstrated that changes in the distribution and expression of AQPs in the intestinal epithelium are associated with gut disorders, including diarrhea [20]. Previous studies showed that piglet diarrhea may be caused by insufficient transport and absorption of water through AQPs in the impaired intestine at the weaning stage [18,21]. Collectively, these findings indicated that AQPs may be potential targets for attenuation of diarrhea. As a common feed additive in pig farming, niacin has been shown to improve the intestinal health of weaned piglets [22,23]. However, the relationship between niacin-mediated alleviation of the weaning stress-induced diarrhea and the changes in AQPs expression, and the underlying mechanism needs further exploration.

G protein-coupled receptor 109A (GPR109A) belongs to the large G protein-coupled receptor family and can be activated by niacin and butyric acid [24]. Recent studies have shown that GPR109 is expressed in intestinal epithelial cells and has a role in regulating microbial metabolism, inhibiting colonic inflammation, and maintaining the integrity of the intestinal barrier [25,26]. Furthermore, supplements with niacin can increase the relative abundance of probiotics in the colon, and alleviate the inflammatory response in the small intestine via GPR109A [23]. However, limited information regarding whether niacin directly regulates the expression of AQPs in weaned piglets via GPR109A.

Therefore, this study aimed to explore whether supplementation with niacin could alleviate diarrhea and intestinal damage of weaned piglets through up-regulation of the expression of AQPs and tight junction proteins induced by GPR109A.

## 2. Results

### 2.1. Effect of Niacin on Intestinal Barrier Function in Weaned Piglet

**Figure 1:**
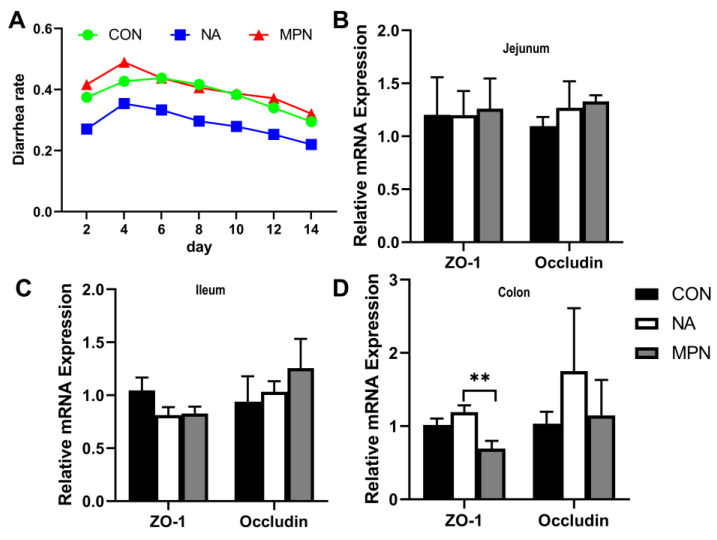
Effect of niacin on the diarrhea rate and mRNA expression of tight junctions in the intestinal mucosa of weaned piglets. (**A**) Diarrhea rate of weaned piglets. (**B**–**D**) QPCR determined the relative mRNA expression of *ZO-1* and Occludin in the jejunal, ileal, and colonic mucosa, respectively. CON, control group; NA, niacin group, MPN, GPR109A receptor blocking group. Values are presented as the mean ± SEM (*n* = 6). ** *p* < 0.01.

Piglets fed a diet supplemented with niacin had a lower diarrhea rate in the 14-day post-weaning trial than those fed with a basic diet or supplementation with the GPR109A antagonist (Figure 1A). Furthermore, compared with piglets fed with an antagonist, pigs in the niacin group had a higher level of *ZO-1* mRNA in the colonic mucosa (Figure 1B–D) (*p* < 0.05).

### 2.2. Effect of Niacin on Intestinal Water Transport in Weaned Piglets

**Figure 2:**
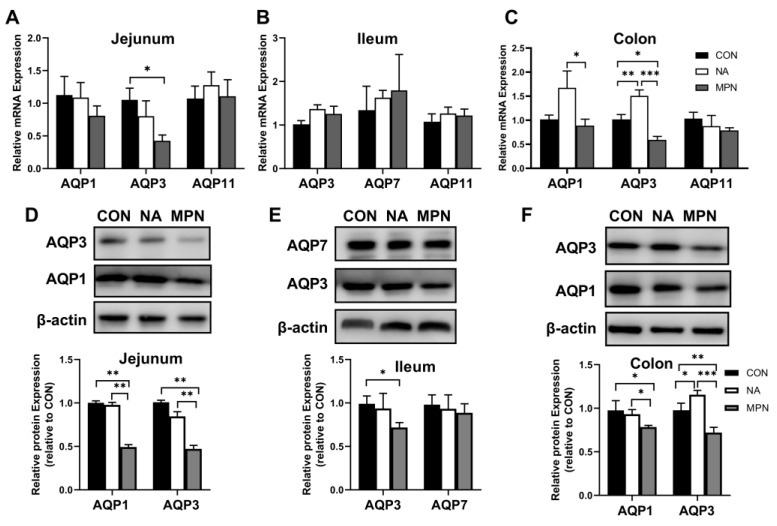
Effect of niacin on the genes and protein expression of AQPs in the intestinal mucosa of weaned piglets. (**A**–**C**) QPCR determined the relative mRNA expression of *AQP1, AQP3, AQP7,* and *AQP11* in the jejunal, ileal and colonic mucosa, respectively. (**D**–**F**) Western blot determined the relative protein expression of AQP1, AQP3, and AQP7 in the jejunal, ileal and colonic mucosa, respectively. CON, control group, NA, niacin group, MPN, GPR109A receptor blocking group. AQP, aquaporin. Values are presented as the mean ± SEM (*n* = 6). * *p* < 0.05, ** *p* < 0.01, *** *p* < 0.001.

Compared with those in the GPR109A receptor blocking group, the mRNA expression of AQP1 and AQP3 were significantly upregulated in the colonic mucosa of the niacin group (Figure 2C) (*p* < 0.05), but no significant difference in the jejunal (Figure 2A) and ileal (Figure 2B) mucosa (*p* > 0.05). At the protein expression level, blocking the GPR109A receptor resulted in a significant down-regulation of AQP1 and 3 protein expression in the jejunal and colonic mucosa (Figure 2D,F, respectively) (*p* < 0.05), but no significant difference was observed in the ileal mucosa (Figure 2E) (*p* > 0.05).

### 2.3. Effect of Niacin on Cell Viability in ETEC-Challenged in IPEC-J2 Cells

**Figure 3:**
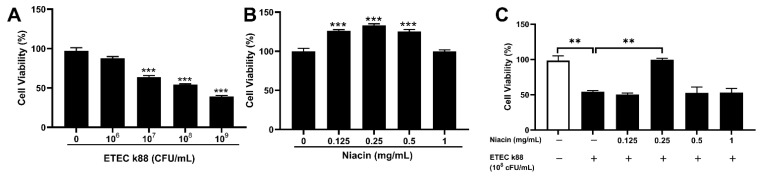
Effect of ETEC K88 or niacin on the cell viability of IPEC-J2 cells. The cell viability was determined by Cell Counting Kit-8 (CCK8) assay in our trial. (**A**) The cell viability after being treated with different concentrations of ETEC K88 for 3.5 h. (**B**) The cell viability after being treated with different concentrations of niacin for 12 h. (**C**) The cell viability after being treated with different concentrations of niacin for 12 h, followed by ETEC K88 (1 × 10^8^ CFU/mL) for 3.5 h. Values are presented as the mean ± SEM (*n* = 3). ** *p* < 0.01, *** *p* < 0.001.

**Figure 4:**
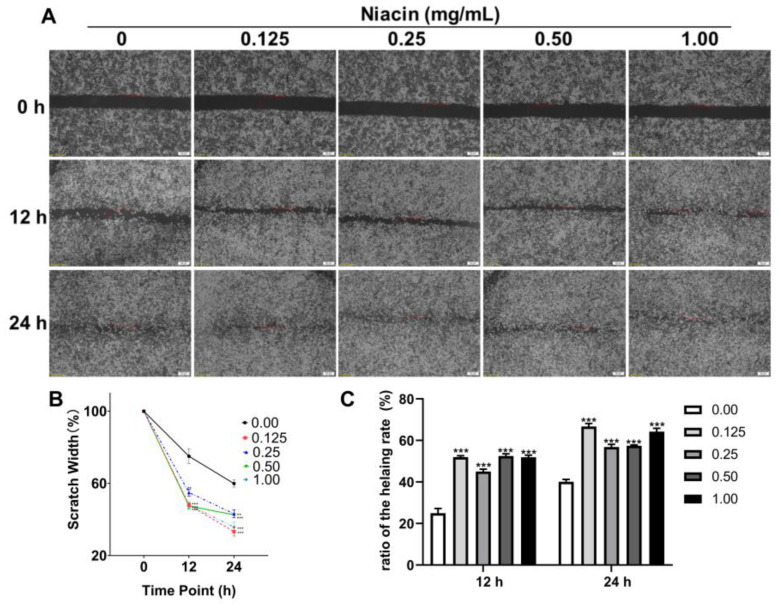
Effect of niacin on the cell migration capacity of IPEC-J2 cells. (**A**) Representative image of the wound-healing assay. The dashed lines indicate wound edges. After scratching, the culture medium was removed, replaced with a fresh medium with or without different concentrations of niacin, and incubated for another 24 h. Scale bar: 50 µm. (**B**) Quantification of migrated scratch width at different time points. (**C**) Quantification of the healing rate. The healing or closure rate is expressed as a ratio of the migration distance (after 24 h) compared with the distance immediately after scratching. Values are presented as the mean ± SEM (*n* = 3). *** *p* < 0.001.

To investigate whether niacin has a protective effect against ETEC-induced apoptosis, we established an intestinal epithelial cell injury model by ETEC infection of IPEC-J2 cells. As shown in Figure 3A, ETEC infection significantly decreased the cell viability after treatment with 1 × 10^7^, 1 × 10^8^, and 1 × 10^9^ CFU/mL *E. coli* k88 for 3.5 h (*p* < 0.05). Then 1 × 10^8^ CFU/mL *E. coli* k88 for 3.5 h was chosen to establish the intestinal epithelial cell injury model on this trail. Meanwhile, supplementation with 0.125, 0.25 and 0.50 mg/mL niacin significantly increased the cell viability after 12 h treatment (Figure 3B) (*p* < 0.05), and promoted the cell migration after 12 and 24 h treatment (Figure 4) (*p* < 0.05). Furthermore, Figure 3C showed that supplemented with 0.25 mg/mL niacin significantly prevented ETEC-induced apoptosis of IPEC-J2 cells (*p* < 0.05).

### 2.4. Effect of Niacin on the mRNA and Protein Expression of Water Transport Proteins in IPEC-J2 Cells

**Figure 5:**
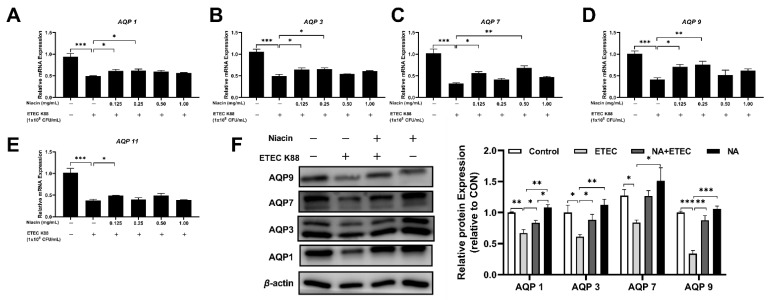
Effect of niacin on the genes and protein expression of AQPs in the IPEC-J2 cells after treatment with ETEC K88. (**A**–**E**) QPCR determined the relative mRNA expression of AQP1, 3, 7, 9, and 11, respectively. (**F**) Western blot determined the relative protein expression of AQP1, 3, 7, and 9 in IPEC-J2 cells, respectively. NA, niacin. AQP, aquaporin. Values are presented as the mean ± SEM (*n* = 3). * *p* < 0.05, ** *p* < 0.01, *** *p* < 0.001.

As shown in Figure 5A–E, compared with the control group, ETEC significantly decreased the mRNA expression of *AQP1*, *AQP3*, *AQP7*, *AQP9*, and *AQP11* (*p* < 0.05). However, supplementation with niacin significantly increased the relative gene expression of the above aquaporins in ETEC-treated cells compared with those in ETEC-treated cells alone (*p* < 0.05). At the protein expression level, ETEC induced a significant down-regulation of AQP1, AQP3, AQP7, and AQP9 expression, while these effects of ETEC were suppressed by 0.25 mg/mL niacin pre-treated cells for 12 h (Figure 5F) (*p* < 0.05).

### 2.5. Niacin Ameliorated ETEC-Induced Cell Apoptosis and Water Transport Impairment via GPR109A in IPEC-J2 Cells

**Figure 6:**
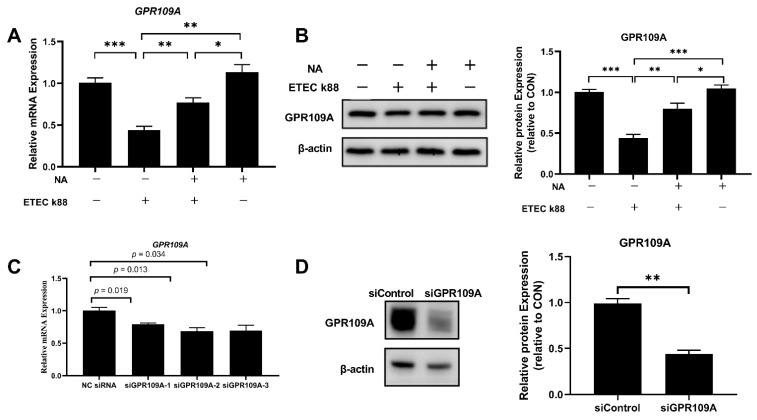
Effect of ETEC K88 or niacin on the mRNA and protein expression of GPR109A in IPEC-J2 cells. (**A**) QPCR determined the relative mRNA expression of GPR109A. (**B**) Western blot determined the relative protein expression of GPR109A. (**C**) QPCR determined the relative mRNA expression of GPR109A in IPEC-J2 cells transfected with control siRNA or siRNA targeting GPR109A. There are three siRNA (siGPR109A-1, siGPR109A-2, siGPR109A-3) designed in the present study, and siGPR109A2 was selected for the following trials. (**D**) Western blot determined the relative protein expression of GPR109A in IPEC-J2 cells transfected with control siRNA or siRNA targeting GPR109A. NA, niacin. GPR109A, G protein-coupled receptor 109A. Values are presented as the mean ± SEM (*n* = 3). * *p* < 0.05, ** *p* < 0.01, *** *p* < 0.001.

**Figure 7:**
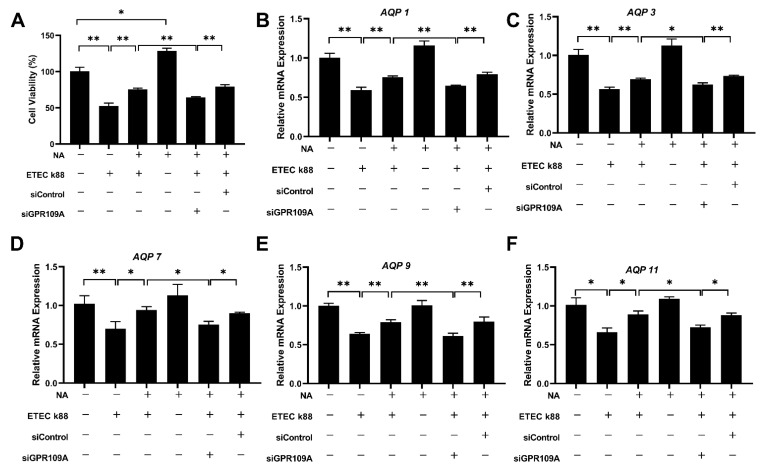
Effect of blocking GPR109A on cell viability and the mRNA expression of *AQPs* in the IPEC-J2 cells. (**A**) CCK8 determined the cell viability in IPEC-J2 cells transfected with control siRNA or siRNA targeting GPR109A. (**B**–**F**) QPCR determined the relative mRNA expression of *AQP1*, *AQP3*, *AQP7*, *AQP9*, and *AQP11* in IPEC-J2 cells transfected with control siRNA or siRNA targeting GPR109A. NA, niacin. AQP, aquaporin. GPR109A, G protein-coupled receptor 109A. Values are presented as the mean ± SEM (*n* = 3). * *p* < 0.05, ** *p* < 0.01.

To determine whether niacin prevented ETEC-induced cell apoptosis and water transport impairment in IPEC-J2 cells through the GPR109A pathway, we examined the genes and protein expression of GPR109A. The results showed that the addition of niacin significantly up-regulated the level of GPR109A gene and protein in ETEC-treated IPEC-J2 cells (Figure 6A,B) (*p* < 0.05). Then we used siRNA to knock down the GPR109A gene and the results showed that the gene and protein expression of GPR109A were decreased (Figure 6C,D) (*p* < 0.05). Moreover, both the cell viability and gene expression of aquaporins (including *AQP1*, *AQP3*, *AQP7*, *AQP9*, and *AQP11*) were significantly decreased (Figure 7) (*p* < 0.05).

## 3. Discussion

Niacin (Pyridine-3-carboxylic acid, C_6_H_5_NO_2_) is a water-soluble vitamin belonging to the vitamin B family, which has been considered to be an odorless white crystalline powder with a feebly acid taste. Previous studies have shown that niacin attenuates the severity of colitis by decreasing colonic MPO activity, TNF-α, and VEGF levels in a guinea pig model [27]. As a feed additive in animal husbandry, an increasing number of reports found that dietary niacin supplement significantly increased the relative abundance of probiotics in the colon, alleviating the inflammatory response in the small intestine [23,28], and increased the function of the intestinal epithelial barrier function [22]. However, little is known about the effect of niacin on the regulation of AQPs expression may be a potential target for the treatment of diarrhea.

A previous study demonstrated that niacin decreased the diarrhea rate of piglets [18]. Consistent with this finding, our results showed that supplementation with niacin reduced the diarrhea rate of piglets after 14 days of weaning. The potential explanation for this finding could be the niacin-mediated up-regulation of colonic ZO-1, AQP1, and AQP3 in weaned piglets. Previous studies have suggested that the physiological functions of AQPs in the intestinal tract involve water transfer and barrier function [29,30]. Inhibition of AQP3 function in the colon caused diarrhea in rats [31]. Similarly, Chao et al. found that altered intestinal permeability was associated with a significantly reduced expression of AQP1 and AQP3, which may result in IBS pathogenesis [32]. In addition, He et al. showed that the protein expression of AQP3, AQP4, and AQP8 in the jejunum and ileum of piglets was decreased by lipopolysaccharide challenge, accompanied by an increased diarrhea index [33]. Consequently, our results implied that niacin reduced the diarrhea of weaned piglets by increasing colonic AQPs expression that enhanced water in colon and intestinal barrier function.

Diarrhea induced by enterotoxigenic Escherichia coli (ETEC) is a major challenge for postweaning piglets, Therefore, this study used an ETEC K88-infected IPEC-J2 cells model to investigate the effect of niacin on diarrhea and the underlying mechanism. Zhu et al. found that ETEC K88 treatment induced lower expression of AQP3, AQP9, and AQP11 in IPEC-J2 cells [11]. Similar to this finding, our study found that the expressions of AQP1, AQP3, AQP7, AQP9, and AQP11 were significantly decreased in IPEC-J2 challenged with ETEC K88. Moreover, niacin prevented ETEC K88-induced a decline in the expression of AQPs, and the cell viability in IPEC-J2 cells. Increasing evidence has demonstrated that AQPs play crucial roles in regulating cell proliferation and repairing intestinal barrier function [10]. A previous study showed that AQP3-deficient mice have lower cell proliferation and more severe colitis compared with wild-type mice [34]. Collectively, our results suggested that niacin-mediated alleviation of the ETEC K88-induced epithelial cell damage may be linked to the alteration of AQPs expression.

Cell migration is a hallmark of wound repair, cancer invasion and metastasis, immune response, angiogenesis, and embryonic morphogenesis [35]. As porcine epithelial cells, after being injured, IPEC-J2 cells will migrate from the edge of the wound to restore the integrity of the skin. In the present study, we indicated that the healing rate was significantly faster and the protective migration capacity was increased in the niacin-treated groups compared to the control. AQPs are considered to be involved in cell migration. Studies by Zhu and Tyteca et al. reported that AQP1 knockout or AQP3 deficiency reduced the migration and phagocytic capacity of macrophages [36,37]. In addition, Hara et al. suggested that AQP3-mediated hydrogen peroxide uptake was required for chemokine-dependent T cell migration in sufficient immune response [38]. Furthermore, Holm et al. reported that after bacterial infection (*Pseudomonas aeruginosa*), macrophage AQP9 expression was increased and the subcellular localization was changed, which was more conducive to migration and phagocytosis [39]. Thus, we suggested that niacin promoted cell migration through increases in the AQPs expression in IPEC-J2 cells. However, information on the potential mechanism by which niacin supplementation regulates AQPs expression use in weaned piglets is limited.

Elucidating how niacin regulates AQPs expression in weaned piglets will help to understand the protective effect of niacin on the treatment of diarrhea induced by weaning stress. Interestingly, we observed significant differences in the expression level of GPR109A between the cells treated with or without ETEC K88 and niacin. GPR109A, G protein-coupled receptor 109A (HM74A or HCA2 in humans), is activated by niacin, butyrate, and hydroxybutyric acid. Increasing evidence has shown that GPR109A plays an important role in inhibiting the inflammatory response and maintaining intestinal mucosal barrier integrity function [40,41]. In our previous study, the results showed that niacin improved the intestinal morphology and the abundance of beneficial bacteria in the colon while alleviating the inflammatory response in the mucosa of the small intestine through a GPR109A-dependent pathway [23]. In this trial, we found that niacin supplementation increased the expression of *GPR109A* mRNA and proteins in ETEC K88-challenged IPEC-J2 cells. Similarly, as a key receptor, supplementation with sodium butyrate also significantly increases the mRNA expression of *GPR109A* in IPEC-J2 cells [42]. Previous studies have also shown that activation of the GPR109A signaling pathway enables sodium butyric acid to repair TNBS-induced inflammatory responses and intestinal epithelial damage [43]. Feng et al. reported that activated GPR109A receptors have an anti-dialysis effect by reducing colonic permeability in weaned piglets [22]. This is consistent with our findings that blocking the GPR109A receptor increased the rate of diarrhea in piglets by reducing colonic ZO-1. However, whether niacin regulates AQPs expression via the GPR109A signaling pathways remains poorly understood. In the present study, the results showed that siRNA GPR109A treatment remarkably reduced cell proliferation, mRNA, and protein expression levels of AQP1, AQP3, AQP7, and AQP9 in IPEC-J2 cells. Hence, we concluded that GPR109A was crucial for up-regulating AQP transcriptional activity.

## 4. Materials and Methods

All animal procedures used in this study were approved by the Animal Care and Use Committee of Guangdong Academy of Agricultural Sciences (authorization number GAASIAS-2016-017). All efforts were made to minimize animal suffering in accordance with the Guidelines for the Care and Use of Animals for Research and Teaching.

### 4.1. Animal Trail Design and Sample Collection

A total of 72 weaned piglets (Duroc × (Landrace × Yorkshire), weaned at 21 d, 6.60 ± 0.05 kg) were randomly assigned to 3 groups for a 14-day feeding trial. Each treatment group included 6 replicate pens and each pen included 4 barrows (*n* = 24/treatment). The 3 groups consist of (1) control group, piglets were fed a basal diet; (2) niacin group, piglets were fed a basal diet supplemented with a 20.4 mg niacin/kg diet; (3) GPR109A receptor blocking group, piglets were fed the basal diet and were also orally given mepenzolate bromide dissolved in ultrapure water, a GPR109A inhibitor, using disposable pipets in a dose of 10 mg/kg piglet’s body weight per day in every morning, the other piglets in the control group and niacin group received the same volume of ultrapure water based on the piglet’s body weight [44,45]. Mepenzolate bromide was obtained from Sigma Chemicals Co. (St. Louis, MO, USA). The piglets had ad libitum access to feed and water at the experimental farm of the Institute of Animal Science, Guangdong Academy of Agricultural Sciences, China. Diets are based on the National Research Council 2012 (NRC 2012) (Table 1). On the morning of the 15th day, One piglet with body weight (BW) closest to the mean BW of each pen was selected and euthanized with sodium pentobarbital (40 mg/kg BW). The intestinal mucosa samples including jejunum, ileum, and colon were scraped with sterile glass microscope slides, then quickly frozen in liquid nitrogen and stored at −80 °C for analysis.

### 4.2. Cell Culture

The intestinal porcine epithelial cell line (IPEC-J2) was kindly supplied by Dr. Guoyao Wu at Texas A and M University (College Station, TX, USA). IPEC-J2 cells were cultured in a serial passage in uncoated plastic culture flasks (100 mm^2^) with DMEM-H medium (Corning, NY, USA) containing 10% FBS (Gibco, Waltham, MA, USA), 100 U/mL penicillin and 100 mg/mL streptomycin (Gibco, Waltham, MA, USA) in an incubator at 37 °C with 5% CO_2_. At the confluence about 80–90%, cells were trypsinized and seeded in six-well cell culture plates with 3 × 10^5^ cells per well. After overnight incubation, cells were starved for 6 h in niacinamide-free DMEM-H. After starvation, the cells were used for subsequent treatment, and 3 replications for each treatment. Plastic culture plates were manufactured by Corning Inc. (Corning, NY, USA).

### 4.3. Bacterial Strains

The ETEC K88 strain was grown in Luria–Bertani broth. After overnight incubation at 37 °C with vigorous shaking at 220 rpm, ETEC K88 was centrifuged at 5000× *g* for 10 min at 4 °C, washed, and resuspended in cold PBS to obtain a final bacterial concentration of approximately 1 × 10^6^, 1 × 10^7^, 1 × 10^8^, 1 × 10^9^ CFU/mL, as described in a previous study [11].

### 4.4. Cell Viability

The viability of cells in our trial was determined by the Cell Counting Kit-8 (CCK8; Beyotime, Jiangmen, China). 100 μL cell suspension (5 × 10^4^ mL^−1^) was added to the well of 96-well micro-culture plates. After overnight culture, the cells were starved for 6 h in 0.2 mL niacinamide-free DMEM-H. After that, the culture medium was removed and then 110 μL of the medium containing 0, 0.125, 0.25, 0.5, 1.0 mg/mLniacin was added and treated for 12 h. After that, cells were treated with 1 × 10^8^ CFU/mL *E. coli* k88 for another 3.5 h. Eight wells containing medium only were used for blanking the reader, another eight wells containing cells and medium were used to determine the control cell survival. Cell viability was measured as previously described [46]. Briefly, cells were incubated in a medium with 10 uL of tetrazolium substrate for 2 h, then the whole 96-well plate was assayed for absorbance at 450 nm and cell viability was calculated using the OD value.

### 4.5. Effect of Niacin on Water Transport Proteins in K88-Challenged IPEC-J2 Cells

IPEC-J2 cells were seeded in six-well cell culture plates with 3 × 10^5^ cells per well. After overnight incubation, cells were starved for 6 h in niacinamide-free DMEM-H. After that, the culture medium was removed and then 2 mL of the medium containing 0, 0.125, 0.25, 0.5, and 1.0 mg/mL niacin was added and treated for 12 h. After that, cells were treated with 1 × 10^8^ CFU/mL *E. coli* k88 for another 3.5 h, and then cells were washed with PBS and harvested for subsequent analysis including qPCR and Western blot analysis.

### 4.6. Cell Migration

IPEC-J2 cells were seeded in six-well cell culture plates with 3 × 10^5^ cells per well. When cells grow to nearly 90% fusion and there is no gap between cells, use the tip of a sterile 200 µL pipette to pass through the cell monolayer to create two separate wounds with consistent width and straight edges. After scratching, PBS was gently washed twice, and the cells were treated with different niacin (0.00, 0.125, 0.25, 0.50, 1.00 mg/mL) for 24 h. Photograph the same scratch three times at 0.12 and 24 h with a microscope and quantify the width of the migrating scratch.

### 4.7. siRNA and Transfection

Seeded in six-well cell culture plates with 3 × 10^5^ cells per well. After overnight culture, the cells were incubated with 20 nM *GPR109A* siRNA or negative control siRNA (NC siRNA) using RNAiMAX and Lipofectamine 2000 (Invitrogen, Grand Island, NY, USA). Firstly, the individual targeted siRNA and plasmid were mixed with Lipofectamine RNAiMAX or lipofectamine 2000 (Invitrogen, Carlsbad, CA, USA), respectively. The RNAiMAX/siRNA mixture was added to IPEC-J2 cells in an antibiotic-free medium and cultured for 8 h. The medium containing siRNA was refreshed with the general medium for another 12 h before subsequent treatments. The sequence of Small interfering RNA (siRNA) targeting pig included siGPR109A-1 (5′-TTA TGT TAA TCA AGA AGC A-3′), siGPR109A-2 (5′-GCA TGT TAA TCA AGA TGC G-3′), siGPR109A-3 (5′-GTA TGT TAA TCA AGA AGT A-3′), control siRNA (5′-UUC UCC GAA CGU GUC ACG UTT-3′). These siRNAs were synthesized by Ribobio (Guangzhou, China).

### 4.8. Quantitative Real-Time PCR (qPCR)

Total cell RNA was extracted using the TRIzol reagent (Beyotime Institute of Biotechnology, Shanghai, China) and reverse transcribed to cDNA using M-MLV reverse transcriptase (Beyotime Institute of Biotechnology, Shanghai, China). qPCR analysis was performed using the SYBR Green PCR Master Mix (Beyotime) with the CFX96 Real-Time PCR System. The data were analyzed following the 2^−∆∆Ct^ method and calculated using *β-actin* as the normalization control. The sequences of primers used are presented in Table 2.

### 4.9. Western Blot Analysis

IPEC-J2 cells and intestinal samples were lysed with radioimmunoprecipitation lysis buffer (Solarbio Life Science. Beijing, China), and then total cellular proteins were gathered. Nuclear proteins were collected by nuclear and cytoplasmic protein extraction kit (Beyotime Institute of Biotechnology). After electrophoresis, the proteins were transferred to polyvinylidene difluoride membranes (Millipore, Bedford, MA, USA). The membranes were incubated with the first antibodies for 12–16 h at 4 °C and then incubated with the secondary antibodies (HRP conjugated anti-rabbit Ab) for 2 h at 21–25 °C. Chemiluminescence signals were detected by ECL western blotting detection reagent (Amersham), and visualized using ChemiScope 3400 (Clinx Science Instruments, Shanghai, China). arbitrary densitometric units for each protein of interest were normalized using those of β-actin.

### 4.10. Statistical Analyses

Data were analyzed using SPSS 20.0 (SPSS Inc., Chicago, IL, USA) and are presented as the mean ± SEM. The results were evaluated by one-way analysis of variance (ANOVA) followed by Tukey’s test. Differences were considered significant at *p* < 0.05.

## 5. Conclusions

In conclusion, the results of the present study indicated that niacin-mediated attenuation of the weaning stress-induced diarrhea and intestinal damage may be linked to the up-regulation of the expression of AQP and tight junction proteins induced by GPR109A.

## Figures and Tables

**Table 1:** Formulation and chemical composition of the basal diet ^1^.

Ingredient	%	Energy and Nutrient Composition	
Corn	34.00	DE, kcal/kg	3526.50
Expanded corn	18.00	ME, kcal/kg	3395.50
Soybean meal	9.50	NE, kcal/kg	2611.30
Expanded soybean	15.00	CP (analyzed), %	19.00
Lactose	2.00	CF (analyzed), %	2.65
Whey powder	8.00	Total phosphorus (analyzed), %	0.68
Fish meal	5.00	Methionine + Cystine, %	0.91
Soybean oil	2.00	Valine, %	1.00
Soybean hull	0.31	Calcium (analyzed), %	0.88
Limestone	0.60	STTD of P, %	0.47
Monocalcium phosphate	1.50		
Lysine 98.5%	0.80		
Methionine	0.30		
L-Threonine	0.30		
Valine	0.13		
L-Tryptophan	0.10		
Salt	0.25		
60% choline chloride	0.15		
Premix ^2^	2.00		
Acidifier	0.05		
Phytase	0.01		
Total	100.00		

^1^ DE, digestive energy; ME, metabolic energy; NE, net energy; CP, crude protein; CF, crude fiber; STTD, Standardized total tract digestible. ^2^ Provided vitamin and mineral premix per kg diet: vitamin A, 2 400 IU; vitamin D_3_, 2 800 IU; vitamin E, 200 IU; vitamin K_3_, 5 mg; vitamin B_12_, 40 μg; vitamin B_1_, 3 mg; vitamin B_2_, 10 mg; pantothenic acid, 15 mg; folic acid, 1 mg; vitamin B_6_, 8 mg; biotin, 0.08 mg; Fe (FeSO_4_·H_2_O), 120 mg; Cu (CuSO_4_·5H_2_O), 16 mg; Mn (MnSO_4_·H_2_O), 70 mg; Zn (ZnSO_4_·H_2_O), 120 mg; I (CaI_2_O_6_), 0.7 mg; and Se (Na_2_SeO_3_), 0.48 mg.

**Table 2:** Primers used for quantitative real-time PCR ^1^.

Primer	Sequence (5′-3′)
Occludin-F	GCACCCAGCAACGACAT
Occludin-R	CATAGACAGAATCCGAATCAC
*ZO-1*-F	GACTCCTTGCTGAATCTGA
*ZO-1*-R	GCACCTCATCATCTTCCAT
*AQP1*-F	TTGGGCTGAGCATTGCCACGC
*AQP1-*R	CAGCGAGTTCAGGCCAAGGGAGTT
*AQP3-*F	CACCTCCATGGGCTTCAACT
*AQP3*-R	TGCCCATTCGCATCTACTCC
*AQP7*-F	AGGCACTTCAGCAGACATCTAA
*AQP7*-R	TGGCGTGATCATCTTGGAGG
*AQP9*-F	TGTCATTGGCCTCCTGATTG
*AQP9*-R	TGGCACAGCCACTGTTCATC
*AQP11*-F	CGTCTTGGAGTTTCTGGCTACC
*AQP11*-R	CCTGTCCCTGACGTGATACTTG
*GPR109A*-F	CCGTCCCACCAGCAGAATCA
*GPR109A*-R	ACCCAGGAGCCCGAACACAA
β-actin-F	CACGCCATCCTGCGTCTGGA
β-actin-R	AGCACCGTGTTGGCGTAGAG

^1^ *AQP1*, aquaporin1; *AQP3*, aquaporin3; *AQP7*, aquaporin7; *AQP9*, aquaporin9; *AQP11*, aquaporin11; GPR109A, G protein-coupled receptor 109A.

## Data Availability

Not applicable.
